# An Evaluation of the Effects of a Non-caffeinated Energy Dietary Supplement on Cognitive and Physical Performance: A Randomized Double-Blind Placebo-Controlled Study

**DOI:** 10.7759/cureus.16178

**Published:** 2021-07-04

**Authors:** Jaime L Tartar, Jose Antonio, Douglas S Kalman, Susan J Hewlings, Joshua Baisley, Mykola Marang, Sarah Flynn, Corey A Peacock

**Affiliations:** 1 Psychology and Neuroscience, Nova Southeastern University, Davie, USA; 2 College of Health Care Sciences, Nova Southeastern University, Davie, USA; 3 The Herbert H & Grace A. Dow College of Health Professions, Central Michigan University, Mt. Pleasant, USA; 4 Clinical Design and Delivery, Nutrasource Pharmaceutical and Nutraceutical Services, Guelph, CAN; 5 Glanbia Research and Development Center, Glanbia Nutritionals, Twin Falls, USA

**Keywords:** attention, cognition, kaempferia parviflora, bacopa monnieri, pomegranate, moringa oleifera, cortisol, saa, time to exhaustion, caffeine

## Abstract

A large and growing body of research shows that non-caffeinated plant-based nutritional supplements can increase cognitive and physical performance. This study aimed to build on this work by investigating the possibility that a specific botanical blend (consisting of *Bacopa monnieri* bacosides, *Kaempferia parviflora* methoxy flavones, pomegranate peel polyphenols, and Moringa oleifera leaf saponins) could improve cognitive and physical performance. To this end, we carried out a randomized, double-blind, placebo-controlled 21-day parallel study on 36 healthy adults. We compared the effects of the botanical blend at baseline to a caffeine and a placebo condition on 1) self-reported alertness, anxiety, and headaches; 2) multiple measures of attention and cognition; 3) physical performance; and 4) stress biomarkers. We found that relative to baseline and compared to the Caffeine and Placebo groups, the botanical blend increased alertness and improved cognitive performance. The cognitive effects were most robust for attention measures. The botanical blend did not improve physical performance on a time to exhaustion (TTE) test. Of note, there was not the expected increase in catecholamine response after the TTE on Day 21, suggesting that long-term botanical blend use decreases the catecholamine stress response of a physical endurance task. In conclusion, we show that, within the confines of this study, a combination of the botanical blend could serve as a safe and effective nutritional supplement to improve cognitive performance.

## Introduction

There is considerable interest in safe and effective nutritional supplements that can improve mental and physical performance. Caffeine (1,3,7-trimethylxanthine) serves as the most widely used supplement that increases performance and alertness [1,2]. Caffeine has demonstrated robust ergogenic effects. Caffeine improves physical performance across a broad range of physical performance measures [3]. Caffeine has also shown extensive improvement in so-called “lower” cognitive processes. This improvement is classically demonstrated by increased vigilance, improved attention, and faster reaction times [4-6]. However, the effect of caffeine on more complex (higher) cognitive processes is less robust. For example, the effects of caffeine on general executive function and decision making are mixed [7]. The scientific consensus regarding basic cognitive functions is that caffeine in doses from 32 to 300 mg (or roughly 0.5-4 mg kg^−1^ for a 75 kg individual) enhances fundamental aspects of cognitive performance, such as attention, vigilance, and reaction time [5,6]. While caffeine has demonstrated physical and mental performance benefits, there are also negative side-effects of caffeine use, which include sleep impairments, anxiety, headaches, and restlessness (jitters). Accordingly, there is growing interest in complementary nutritional supplements to increase performance.

Plant-based supplements without caffeine have shown great promise in increasing mental and physical performance, without the unwanted side effects of caffeine. The current study was specifically interested in the established effects of a botanical blend since it includes ingredients that have previously been shown to increase physical performance measures - a plant-based blend of *Kaempferia parviflora* methoxy flavones, pomegranate peel polyphenols, and Moringa oleifera leaf saponins. This blend was previously shown to acutely increase nitrate (NO_3_^−^) and nitrite (NO_2_^−^) as well as nitric oxide (NO) levels, which promote increased vasodilation and blood flow [8]. These effects should translate into increased physical performance through enhanced physical endurance. Indeed, long-term (22-day) supplementation with this blend of ingredients was shown to significantly reduce oxidative stress to the muscles and tissues during exercise and significantly increase performance (distance run on a treadmill) on a time to exhaustion (TTE) test [9].

The botanical blend was also of interest in the current study because it includes extract from *Bacopa monnieri*. There is growing evidence that a plant-based extract from *Bacopa monnieri* confers considerable cognitive benefits. A meta-analysis concluded that *Bacopa monnieri* extract improves cognition, especially attention measures [10]. Indeed,* Bacopa monnieri* extract has recently been proposed as a potential adjuvant treatment for Alzheimer's disease and related neurodegenerative diseases [11,12]. The mechanisms through which *Bacopa monnieri* extract improves cognition are thought to be due to a combination of pathways including an increase in antioxidant neuroprotection, a decrease in acetylcholinesterase activity, a reduction in amyloid β build-up, improved cerebral blood flow, as well as modulation of indolamine and catecholamine neurotransmitters (including acetylcholine) [13].

Given the demonstrated performance benefits of the combination of ingredients in the botanical blend, we hypothesized that it would confer both physical as well as cognitive performance benefits, without associated adverse consequences (e.g. anxiety, headache, stress, or jitters). To address this question, we investigated the acute and long-term (21-day) effects of botanical blend on a series of cognitive and physical performance measures compared to caffeine and placebo. We also tested the extent to which these three treatment conditions (botanical blend, caffeine, and placebo) influenced alertness, anxiety, and headache. Finally, we assessed the effects of the supplement conditions on stress biomarkers before and after cognitive and physical performance testing.

## Materials and methods

This was a randomized, double-blind, placebo-controlled 21-day parallel study. The study evaluated the effects of a non-caffeinated energy dietary supplement compared to green tea caffeine extract and a placebo on cognitive and physical performance in healthy, physically active adults. The botanical blend was composed of *Bacopa monnieri *extract, moringa saponins, and polyphenols and methoxyflavones derived from both pomegranate peel and black ginger extracts. 

Participants were randomized in a 1:1:1 ratio to one of the three following investigational groups: 1) the Botanical blend, 2) Caffeine (green tea caffeine extract [170 mg; to deliver 34 mg caffeine {20% natural caffeine}]), or 3) Placebo (inactive ingredients: dextrose, alkalized cocoa, natural flavors [chocolate, caramel, vanilla cream], silica, inulin, sucralose). All investigational products were provided by Glanbia Nutritionals (USA) along with the corresponding certificates of analysis. The flavored powder stick packs were blended by Uckele Health & Nutrition (USA). The participants underwent one screening session and four testing sessions (Test Day 1 for baseline assessments, Test Days 7 and 14 for cognitive testing, and Test Day 21 for end of study assessments). Products were administered during the visits and daily at home on non-test days.

Participants

Participant testing was carried out in accordance with the Declaration of Helsinki and a study protocol approved by the Institutional Review Board at Nova Southeastern University (NSU, IRB 2019-563). All participants received a verbal explanation of the study procedures and signed an NSU IRB-approved written informed consent form. Testing occurred between January 2020 and January 2021. Thirty-six participants (12 participants per group, 22 males, 14 females) were randomized to one of the three study groups. Participants had a healthy body mass index (BMI, average in the healthy weight category <25 kg/m^2^) and low percent body fat (PBF, healthy weight category <25%). All participants were self-reported caffeine users. Refer to Table 1 for additional information on overall participant characteristics. All participants underwent screening procedures to assess eligibility for study participation, which included a review of medical history, anthropometrics, physical fitness readiness questionnaire, a sleep questionnaire (The Pittsburg Sleep Quality Index), heart rate and blood pressure, body weight, and body composition.

**Table 1:** Participant Demographic Characteristics. Participant demographic characteristics include age, height, weight, BMI, and PBF. Means and standard deviations are shown. BMI, body mass index; PBF, percent body fat.

Age (years)	Height (cm)	Weight (kg)	BMI (kg/m^2^)	PBF (%)
27.50 (8.33)	175.27 (16.09)	73.32 (14.01)	24.83 (4.20)	22.99 (9.30)

Procedures

Participants were randomized on Test Day 1 and were educated on the study process and how to take their assigned study product dose daily with the supplied study product (instructions were also on each packet). On Days 1 and 21, all participants underwent the following sequence of events: resting blood pressure and heart rate, saliva collection (for cortisol and salivary alpha amylase quantification), mental energy assessment, cognition testing, onsite supplement administration, 1-h wait, saliva collection, mental energy assessment, cognition testing, and physical performance testing (TTE). On Test Days 7 and 14, all participants underwent the following sequence of events: resting blood pressure and heart rate, on-site supplement administration, 1-h wait, saliva collection, and cognition testing. All testing occurred in the afternoon between 1300 and 1800 h.

Mood, Alertness, and Physical Sensation Scale

Participants rated mood and physical sensation states according to how they were feeling “at the moment” one at a time on the computer monitor using E-prime software (Psychology Software Tools, Inc., Pittsburgh, PA, USA). The Mood, Alertness, and Physical Sensation Scale (MAPSS) included 19 items (single or groups of words/descriptors) describing moods and physical sensations, which were rated on a nine-point scale anchored at the left-hand end with "not at all" and the right-hand end with "extremely". Three subscales were assessed based on Alertness, Anxiety, and Headache [14].

Cognitive testing

A series of cognitive testing instruments from the National Institutes of Health (NIH) Cognition Toolbox [15,16] and the Psychomotor Vigilance Test (PVT) from Joggle Research (Joggle Research Inc., Seattle, WA, USA) [17] were utilized to offer a comprehensive assessment of cognitive processing. The assessments were run on an iPad app from the NIH Toolbox and Joggle. The tests have been designed and validated for use in clinical assessment and clinical trials. Each measure has excellent test-retest reliability (these are widely used instruments with reported Spearman's correlation range from 0.86 to 0.92) [16,17]. The individual assessments from the NIH toolbox were measured independently with a T (normalized) score. The PVT outcome measures were reaction time and errors. The total testing time for the neurobehavioral assessments was approximately 20 min. The Flanker Inhibitory Control and Attention Test (Flanker) measured the participant’s attention and inhibitory control. The Pattern Comparison Processing Speed Test (Processing Speed) assessed the participant’s ability to quickly process information. The Dimensional Change Card Sort Test (DCCS) assessed executive function. The PVT measured the participant’s behavioral alertness.

Biomarkers

Saliva samples were collected from each participant via passive drool into polyethylene tubes at three time points (pre-dose [baseline], 60 min after the dosing of the supplement, and after the TTE test) on Days 1 and 21. Immediately after collection, sample tubes were stored in a -20°C freezer. On the day of analyses, the saliva samples were thawed, vortexed, and centrifuged at 3,000 rpm (0.9 g) for 15 min. Saliva samples were run in duplicate. Cortisol was quantified via a human enzyme immunoassay kit and salivary alpha-amylase (sAA) was quantified via a kinetic reaction kit per the manufacturer’s instructions (Salimetrics, LLC, USA). The samples were immediately read in a BioTek ELx800 plate reader (BioTek Instruments, Inc., USA) at 450 nm with a correction at 630 nm for cortisol and at 405 nm for sAA. All samples were within the detection ranges indicated in the immunoassay kits (Salimetrics, LLC, USA), and the variations of sample readings were within the expected limits. Final concentrations for the biomarkers were generated by interpolation from the standard curve in U/mL for sAA and μg/dL for cortisol. The coefficient of variation for both assays was in acceptable limits and kit sensitivity ranges can be found on the manufacturer's website (Salimetrics, LLC, USA). 

Exercise performance - treadmill time to exhaustion test

All participants were first fitted with a Polar® heart rate sensor (Bethpage, NY, USA). All participants were allowed to warm up for 3-min blocks (6 total minutes) on a treadmill. Block 1 was walking for 3 min at 1.7 miles per hour (MPH) at 0% incline, followed by walking at 3.5 MPH at 1% incline for 3 min. After the warmup was concluded, participants were asked to run on the treadmill at 6.0 MPH (females) or at 7.0 MPH (males). The incline of the treadmill was increased each minute by 1% until participants indicated that they could no longer run safely. If a participant reached maximum incline (12%) they stayed at that incline until exhaustion was reached. The number of minutes and seconds completed was documented by the study researchers; this number was considered the TTE score. Heart rate was recorded at the time the participant stopped running.

Statistical analyses

Repeated-measures (RM) analysis of variance (ANOVA) was used to examine the effect of treatment and session on each outcome variable. Group status (treatment group) served as a between-subjects factor while testing session served as the within-subject factor. We carried out planned comparisons [18,19] on test variables using paired-samples t-tests. In instances where the sphericity assumption was not met, the reported p-values associated with the F statistics were adjusted via the Greenhouse-Geisser correction. All calculations were conducted using an SPSS statistical package (version 26, IBM, Armonk, NY, USA). All reported p-values are two-tailed with an a priori significance level of p <0.05.

## Results

Group participant characteristics

In general, the groups were matched on age and anthropometrics (even across the groups). There was a significant between-group difference in body weight [F(2,33) = 5.09, p =0.01]. Follow-up Bonferroni tests showed that this difference was due to the Placebo group weighing significantly more than the Botanical blend group (p = 0.03). Further inspection of the data revealed three larger males in the control group that drove this difference; however, the non-significant differences in BMI and PBF reflect the overall health and anthropometric similarities. See Table 2.

**Table 2:** Participant Characteristics by Group Means and Standard Deviations. Asterisks (*) indicate significant difference from the Botanical blend group, * = p <0.05. BMI, body mass index; PBF, percent body fat.

Group	Age (years)	Height (cm)	Weight (kg)	BMI (kg/m^2^)	PBF (%)
Botanical blend	24.33 (7.67)	169.73 (7.62)	68.43 (9.67)	25.60 (5.09)	24.47 (10.57)
Caffeine	27.83 (7.98)	174.14 (11.48)	68.11 (10.21)	23.11 (2.69)	22.78 (7.83)
Placebo	30.33 (8.87)	181.94 (23.47)	82.60 (16.91)*	25.78 (4.27)	21.73 (9.91)

Mood, Alertness, and Physical Sensations Scale

Mental alertness was assessed via the MAPSS. A 3 x 4 (Group x Session) RM ANOVA did not reveal a significant Group effect F(1,33) = 1.32, p = 0.28, a significant main effect of Session F(3,99) = 0.75, p = 0.53 nor a significant Group x Session interaction F(6,99) = 2.04, p = 0.07. However, planned paired-samples t-tests showed that relative to baseline (Day 1 Test 1), the Botanical blend group showed a significant increase in alertness post-dose (Day 1 Test 2), t(11) = 2.84, p = 0.02 and at baseline Day 21 (Day 21 Test 1), t(11) = 3.01, p = 0.01. There were no significant within-group differences comparing Day 21 Time 1 with Day 21 Time 2 (Table 3).

**Table 3:** Alertness (MAPSS). Asterisks (*) indicate significant difference from baseline Day 1 (Day 1, Test 1), * = p <0.05. Note: Units are self-report scores on a nine-point scale. MAPSS, Mood, Alertness, and Physical Sensations Scale.

	Botanical blend		Caffeine		Placebo	
	Mean	SD	Mean	SD	Mean	SD
Day 1 Test 1	6.84	1.05	6.51	1.41	7.19	0.86
Day 1 Test 2	7.24*	1.23	6.75	1.69	7.03	0.83
Day 21 Test 1	7.58*	0.84	6.16	1.81	6.42	1.49
Day 21 Test 2	7.34	1.48	6.54	1.63	6.75	1.42

**Figure 1:**
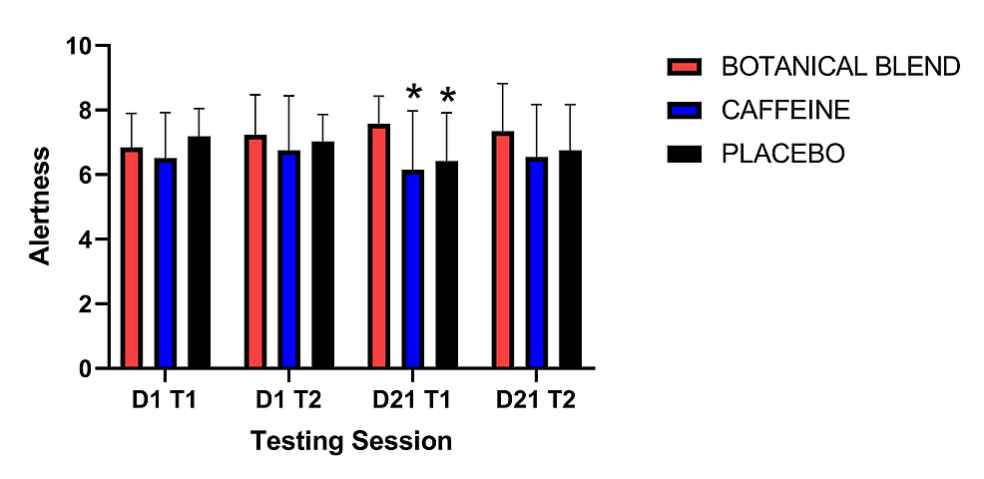
MAPSS Alertness. MAPSS alertness subscale. Independent-samples t-tests comparing groups to each other at each testing session. Asterisks (*) indicate significant difference from the botanical blend, p < 0.05. MAPSS, Mood, Alertness, and Physical Sensation Scale.

Follow-up independent-samples t tests showed that in the third MAPSS assessment (Day 21 Test 1), the Botanical blend group rated themselves as significantly more alert (mean = 7.58, SD = 0.84) than the Caffeine group (mean = 6.16, SD = 1.81), t(22) = 2.48, p = 0.02 and the Placebo group (mean = 6.42, SD = 1.49), t(22) = 2.36, p = 0.03 (Figure 1). 

A 3 x 4 (Group x Session) RM ANOVA for the MAPSS anxiety subscale did not reveal a significant Group effect F(1,33) = 0.26, p = 0.77. There was a significant main effect of Session F(3,99) = 3.99, p = 0.01 as well as a significant Group x Session interaction F(6,99) = 1.96, p = 0.02. Paired-samples t-tests showed that relative to baseline (Day 1 Test 1), the Caffeine group showed a significant decrease in anxiety post-dose Day 21 (Day 21 Test 2), t(11) = 2.82, p = 0.02. The Placebo group showed a significant decrease in anxiety on Day 21 at baseline (Day 21 Test 1), t(11) = 2.38, p = 0.04 and post-dose (Day 21 Test 2), t(11) = 4.46, p = 0.001. A paired-samples t-test between post-dose Day 1 (Day 21 Test 2) and post-dose Day 21 (Day 21 Test 2) also showed a significant decrease in anxiety in the Placebo group, t(11) = 2.22, p = 0.049 (Table 4). There were no between-group differences at any testing session (all p’s >0.05).

**Table 4:** Anxiety (MAPSS). Asterisks (*) indicate significant difference from baseline Day 1 (Day 1, Test 1), ** = p <0.01. Double dagger (‡) indicates significant difference from post-dose Day 1 (Day 1, Test 2),  p < 0.05. Note: Units are self-report scores on a nine-point scale. MAPSS, Mood, Alertness, and Physical Sensation Scale.

	Botanical blend		Caffeine		Placebo	
	Mean	SD	Mean	SD	Mean	SD
Day 1 Test 1	1.97	1.26	2.53	1.18	2.37	0.96
Day 1 Test 2	1.67	1.35	2.18	1.16	1.8	0.72
Day 21 Test 1	2.2	1.42	1.95	0.89	1.78^*^	0.71
Day 21 Test 2	2.2	1.43	1.70^*^	0.97	1.37^**‡^	0.54

A 3 x 4 (Group x Session) RM ANOVA for the MAPSS headache subscale did not reveal a significant Group effect F(1,33) = 186, p = 0.17, a significant main effect of Session F(3,99) = 0.18, p = 0.91, nor a significant Group x Session interaction F(6,99) = 0.63, p = 0.70. Follow-up paired-samples t-tests did not show any significant between-group difference (all p’s >0.05).

Vigilant attention reaction (response) time was assessed via the PVT. A 3 x 6 (Group x Session) RM ANOVA for response time did not reveal a significant Group effect F(1,33) = 1.07, p = 0.36, a significant main effect of Session F(5,165) = 2.01, p = 0.08, or a significant Group x Session interaction F(10,165) = 1.76, p = 0.07. Paired-samples t-tests showed that the Placebo group had a significantly slower reaction time after dosing in session 1 (Day 1 Test 2), t(11) = 2.33, p = 0.04. A paired-samples t-test comparing Day 21 Test 1 to Day 21 Test 2 showed that the Caffeine group had a significantly slower reaction time on Day 21 Test 2, t(11) = 2.35, p = 0.04. The Caffeine group also had a significantly slower reaction time comparing post-dose Day 21 (Day 21 Test 2) to Day 1 post-dose (Day 1 Test 2), t(11) = 2.53, p = 0.03. There were no significant within-group differences comparing Day 21 Time 1 with Day 21 Time 2 (Table 5).

**Table 5:** Psychomotor Vigilance Task Reaction Time. Asterisks (*) indicate significant difference from baseline Day 1 (Day 1, Test 1), * = p < 0.05. Double Dagger (‡) indicates significant difference from post-dose Day 1 (Day 1, Test 2),  p < 0.05. Note: Units are in milliseconds (higher score indicates slower response time).

	Botanical blend		Caffeine		Placebo	
	Mean	SD	Mean	SD	Mean	SD
Day 1 Test 1	282.56	19.73	279.36	53.78	255.87	27.79
Day 1 Test 2	273.48	36.22	274.68	55.96	273.86^*^	45.47
Day 7	274.24	26.09	284.92	46.99	261.5	46.46
Day 14	274.24	26.09	283.3	50.07	265.84	48.18
Day 21 Test 1	274.32	27	299.45^*^	52.71	255.59	41.08
Day 21 Test 2	281.78	27.94	307.93^‡^	73.39	272.3	69.78

**Figure 2:**
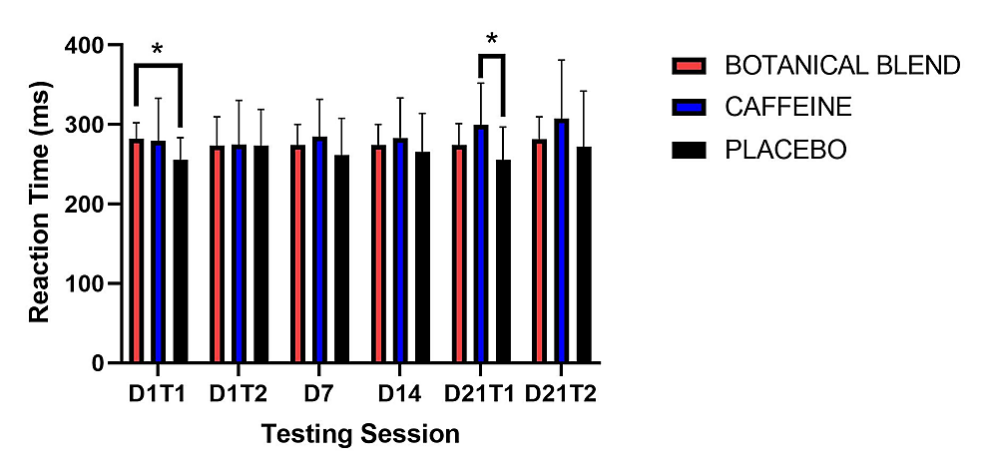
PVT Reaction Time. PVT reaction time measures between groups (higher values reflect slower responses). Asterisks (*) indicate p <0.05. PVT, Psychomotor Vigilance Task.

As shown in Figure 2, follow-up independent-samples t-tests showed that for reaction time in the first assessment (Day 1 Test 1), the Botanical blend group (mean = 282.56, SD = 19.73) had a slower reaction time than the Placebo group (mean = 255.87, SD = 27.79), t(22) = 2.713, p = 0.01. In the fifth PVT assessment (Day 21 Test 1), the Caffeine group had a significantly slower reaction time (mean = 299.45, SD = 52.71) than the Placebo group (mean = 255.52, SD = 41.08), t(22) = 2.27, p = 0.03.

**Figure 3:**
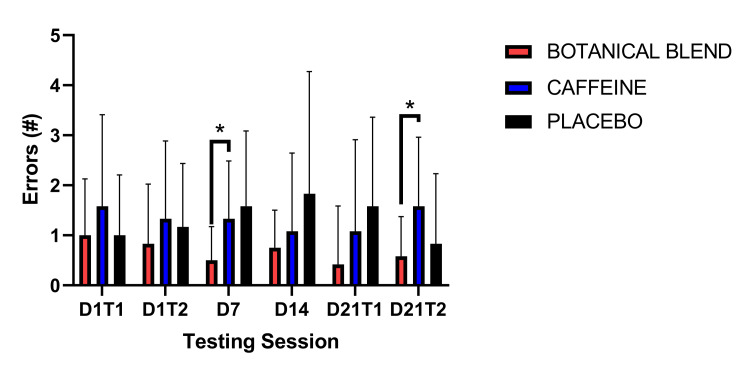
PVT Errors. PVT error measures between groups. Asterisks (*) indicate p <0.05. PVT, Psychomotor Vigilance Task.

PVT errors were assessed via a 3 x 6 (Group x Session) RM ANOVA. There was not a significant Group effect F(1,33) = 0.25, p = 0.25, a significant main effect of time F(5,165) = 0.27, p = 0.93, or a significant Group x Session interaction F(10,165) = 1.28, p = 0.24. Paired-samples t-tests (comparing each assessment time to Day 1 Time 1 and comparing Day 21 Test 1 to Day 21 Test 2) showed that there were no significant within-group differences comparing Day 21 Time 1 with Day 21 Time 2. There were no significant within-group differences comparing Day 21 Time 1 with Day 21 Time 2. There were no significant within-group differences comparing Day 1 Time 2 with Day 21 Time 2 (all p’s >0.05; data not shown). As shown in Figure 3, follow-up independent-samples t-tests for errors showed that on the third assessment (Day 7), the Botanical blend group had significantly fewer errors (mean = 0.50, SD = 0.67) than the Caffeine group (mean = 1.33, SD = 1.16), t(22) = 2.16, p = 0.04. The same pattern was observed on sixth assessment (Day 21 Test 2); the Botanical blend group had significantly fewer errors (mean = 0.58, SD = 0.79) than the Caffeine group (mean = 1.58, SD = 1.38), t(22) = 2.16, p = 0.04.

Flanker Measure of Inhibitory Control and Attention

The NIH Toolbox was used to examine Attention and Inhibitory Control (Flanker). A 3 x 6 (Group x Session) RM ANOVA revealed a significant main effect of time F(5,165) = 7.97, p < 0.001. However, there was not a significant Group effect F(1,33) = 2.23, p = 0.12 nor a significant Group x Session interaction F(10, 165) = 0.93, p = 0.51. Paired-samples t-tests comparing each group to Baseline (Day 1 Test 1) showed that for the Botanical blend group, there was a significant increase in Flanker performance on Day 14 t(11) = 2.88, p = 0.02, Day 21 Test 1 t(11) = 3.56, p = 0.004, and Day 21 Test 2 t(11) = 3.60, p = 0.004. For the Caffeine group, there was a significant improvement in performance on Day 21 Test 1 t(11) = 2.33, p = 0.04. For the Placebo group, there was a significant improvement in performance on Day 7 t(11) = 2.54, p = 0.03 and Day 21 Test 2 t(11) = 2.40, p = 0.04. Compared to post-dose on Day 1 (Day 1 Time 2), there was a significant improvement in Flanker performance on Day 21 post-dose (Day 21 Time 2) for the Botanical blend group [t(11) = 2.25, p = 0.046] and the Placebo group [t(11) = 2.35, p = 0.038]. There were no significant within-group differences comparing Day 21 Time 1 with Day 21 Time 2 (Table 6).

**Table 6:** Flanker Inhibitory Control and Attention Test. Asterisks (*) indicate significant difference from baseline (Day 1, Test 1), * = p <0.05 ** = p <0.01. Double dagger (‡) indicates significant difference from post-dose Day 1 (Day 1, Test 2),  p < 0.05. Note: Units are in standardized (T) scores.

	Botanical blend		Caffeine			Placebo		
	Mean	SD	Mean		SD	Mean		SD
Day 1 Test 1	58.33	11.93	54.58		10.66	58.33		7.43
Day 1 Test 2	62.92	8.52	54.33		10.37	58.83		8.37
Day 7	62.33	9.65	57.5		12.53	65.00^*^		8.89
Day 14	66.25^*^	7.34	58.17		10.1	62.67		10.82
Day 21 Test 1	67.17^**^	8.06	60.75^*^		9.29	62.67		9.73
Day 21 Test 2	67.83^**^^‡^	7.17	59.5		10.67	64.17^*^^‡^		7.02

**Figure 4:**
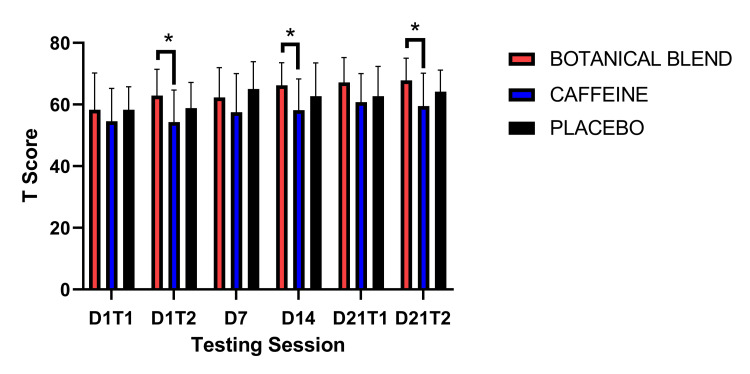
Flanker Inhibitory Control and Attention Test. Units are in standardized (T) scores. Asterisks (*) indicate p <0.05.

Follow-up independent-samples t-tests for the Flanker test showed that at three time points the Botanical blend group scored significantly higher than the Caffeine group. This was on Day 1 Test 2: Botanical blend (mean = 62.92, SD =8.52) and Caffeine (mean = 54.33, SD = 12.53), t(22) = 2.22, p = 0.04; Day 14: Botanical blend (mean = 66.25, SD = 7.34) and Caffeine (mean = 58.17, SD = 10.10), t(22) = 2.24, p = 0.04; Day 21 Test 2: Botanical blend (mean = 67.83, SD = 7.17) and Caffeine (mean = 59.5, SD = 10.67), t(22) = 2.25, p = 0.04]. See Figure 4.

Dimensional Change Card Sort Test

**Figure 5:**
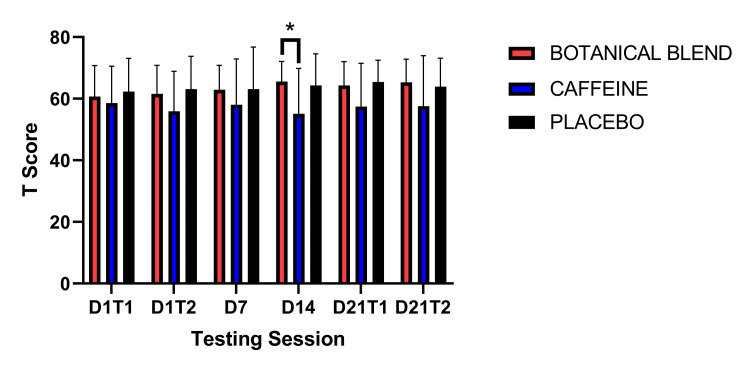
Dimensional Change Card Sort Test. Units are in standardized (T) scores. Asterisk (*) indicates p <0.05.

The NIH Toolbox was used to examine executive function (DCCS Test). A 3 x 6 (Group x Session) RM ANOVA did not reveal a significant Group effect F(1,33) = 1.68, p = 0.20, a significant main effect of time F(5,165) = 0.87, p = 0.50, nor a significant Group x Session interaction F(10, 165) = 0.83, p = 0.60. There were no significant within-group effects at any time point following baseline on Day 1 (Day 1 Test 1), on Day 21 Test 1 compared to Day 21 Test 2, or on Day 21 Test 2 compared to Day 21 Test 2 (all p’s >0.05). Data are not shown. Follow-up independent-samples t-tests showed that on Day 14 the Botanical blend group (mean = 65.58, SD = 6.53) scored significantly higher than the Caffeine group (mean = 55.08, SD = 14.74), t(22) = 2.25, p = 0.04 on the fourth testing session (Day 14, see Figure 5).

Pattern Comparison Processing Speed Test

NIH Toolbox was used to examine Pattern Comparison Processing Speed Test. A 4 x 6 (Group x Session) RM ANOVA revealed a significant main effect of Session F(5,165) = 7.86, p = < 0.001. However, there was not a significant group effect F(1,33) = 1.62, p = 0.21 or a significant Group x Session interaction F(10, 165) = 0.89, p = 0.55.

 Paired-sample tests showed that for the Botanical blend group, there was a significant increase in performance on Day 21 Test 2 t(11) = 2.22, p = 0.048. For the Caffeine group there was a significant increase in performance on Day 1 Test 2, t(11) = 2.66, p = 0.02; Day 14, t(11) = 2.41, p = 0.03; and Day 21 Test 2, t(11) = 2.64, p = 0.02. For the Placebo group there was a significant increase in performance on Day 7, t(11) = 2.59, p = 0.03 and Day 14, t(11) = 2.66, p = 0.02. There were no significant within-group differences comparing Day 21 Time 1 with Day 21 Time 2. There were no significant within-group differences comparing Day 1 Time 2 with Day 21 Time 2 (Table 7).

**Table 7:** Pattern Comparison Processing Speed Test. Asterisks (*) indicate significant difference from baseline (Day 1, Test 1), * = p <0.05. Note: Units are in standardized (T) scores.

	Botanical blend		Caffeine		Placebo	
	Mean	SD	Mean	SD	Mean	SD
Day 1 Test 1	66.83	5.01	63.75	12.29	69.25	9.09
Day 1 Test 2	68.17	6.41	72.08^*^	5.25	72.83	5.11
Day 7	69.58	6.19	70.17	8.54	73.50^*^	6.52
Day 14	70	5.19	71.33^*^	7.81	74.42^*^	4.81
Day 21 Test 1	70.33	4.03	69.5	10.94	74.25	4.67
Day 21 Test 2	71.17^*^	4.49	72.08^*^	7.03	74.33	4.14

**Figure 6:**
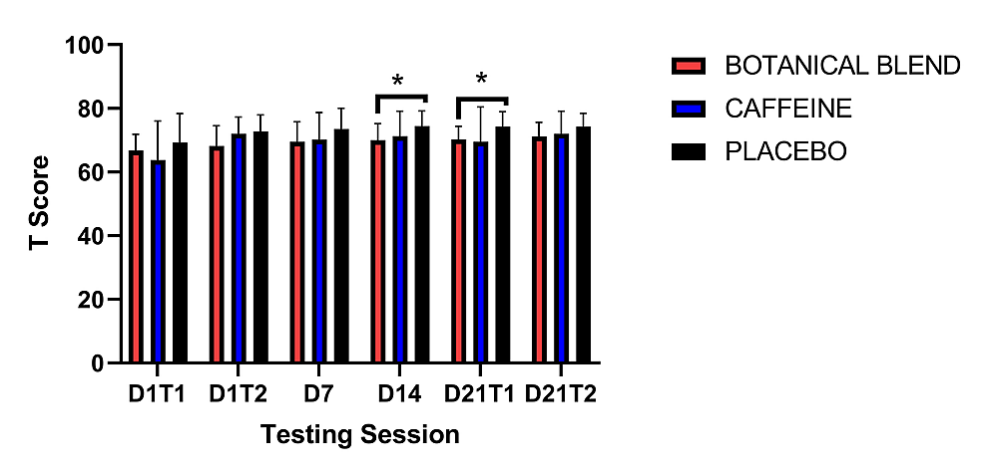
Pattern Comparison Processing Speed Test. Units are in standardized (T) scores. Asterisks (*) indicate p <0.05.

Follow-up independent-samples t-tests showed that at two time points (Days 14 and 21) the Botanical blend group had significantly lower performance on processing speed than the Placebo group [Day 14: the Botanical blend (mean = 70.00, SD =5.19), Placebo (mean = 74.42, SD = 4.81), t(22) = 2.16, p = 0.04; Day 21 Test 1: the Botanical blend (mean = 70.33, SD = 4.03), Placebo (mean = 74.25, SD = 4.67), t(22) = 2.20, p = 0.04] (Figure 6). 

Physical performance: time to exhaustion test

There were no significant differences in percent changes on TTE time or heart rate (all p’s >0.05). See Table 8 for percent differences, Table 9 for raw score run time, and Table 10 for raw score maximum heart rate on the TTE test. Independent and paired-samples t-tests were performed on raw score TTE times and maximum heart rates There were no significant within- or between-group differences (all p’s >0.05).

**Table 8:** TTE Test: Percent Change in Running Time and Maximum HR. TTE, time to exhaustion; HR, heart rate.

	Botanical blend		Caffeine		Placebo	
	Mean	SD	Mean	SD	Mean	SD
TTE time	1.44	10.49	-2.3	19.39	1.91	16.82
TTE max HR	0.75	6.43	0.74	17.15	6.82	13.52

**Table 9:** Time to Exhaustion Test: Running Time in Seconds.

	Botanical blend		Caffeine		Placebo	
	Mean	SD	Mean	SD	Mean	SD
Day 1	301	172.59	287.83	194.67	387.83	240.72
Day 21	310.58	180.93	295.58	202.61	414.25	265.89

**Table 10:** Time to Exhaustion Test: Maximum Heart Rate (Beats Per Minute).

	Botanical blend		Caffeine		Placebo	
	Mean	SD	Mean	SD	Mean	SD
Day 1	180.5	12.09	171.67	29.19	158.17	31.98
Day 21	182.08	9.6	173.5	15.87	170.58	28.42

Cortisol

A 3 x 8 (Group x Session) RM ANOVA did not reveal a significant Group main effect F(1,33) = 0.57, p = 0.57, a main effect of Session F(7,231) = 1.58, p = 0.14, or a significant Group x Session interaction F(14,231) = 0.77, p = 0.70. Paired-sample t-tests comparing each timepoint to baseline (Day 1, Time 1) showed a significant increase in cortisol for the Botanical blend group during baseline collection on Day 21 (Day 21 Time 1), t(11) = 2.31, p = 0.04 and a significant decrease in cortisol in the Caffeine group 1 h after dosing on Day 1 (Day 1 Test 2) t(11) = 2.50, p = 0.03. There were no significant within-group differences comparing Day 21 Time 1 with Day 21 Time 2 (Table 11). There were no significant within-group differences comparing Day 1 Time 2 with Day 21 Time 2. Independent-samples t-tests did not reveal any significant group differences in cortisol at any testing time point (all p’s <0.05).

**Table 11:** Cortisol. Asterisks (*) indicate significant difference from baseline (Day 1, Test 1), * = p <0.05 Note: Units are µg/dL.

	Botanical blend		Caffeine		Placebo	
	Mean	SD	Mean	SD	Mean	SD
Day 1 Test 1	0.1	0.06	0.27	0.28	0.34	0.61
Day 1 Test 2	0.1	0.04	0.17^*^	0.24	0.15	0.23
Day 1 post-TTE	0.16	0.09	0.15	0.14	0.17	0.2
Day 7	0.11	0.06	0.35	0.85	0.39	0.94
Day 14	0.09	0.08	0.16	0.12	0.2	0.33
Day 21 Test 1	0.19^*^	0.15	0.47	1.03	0.26	0.38
Day 21 Test 2	0.12	0.08	0.42	1.03	0.2	0.29
Day 21 post-TTE	0.15	0.1	0.28	0.6	0.21	0.15

Salivary alpha amylase

 A 3 x 8 (Group x Session) RM ANOVA did not reveal a significant Group main effect F(1,33) = 0.1.15, p = 0.33. There was a significant main effect of Session F(7,231) = 12.63, p < 0.001. There was not a significant Group x Session interaction F(14, 231) = 0.71, p = 0.85. Paired-sample t-tests comparing each timepoint to baseline (Day 1, Time 1) showed a significant increase in sAA for the Botanical blend group post-TTE on Day 1, t(11) = 2.31, p = 0.03 and at baseline on Day 21 (Day 21, Test 1), t(11) = 4.38, p = 0.04. The Caffeine group showed a significant increase in sAA post-TTE on Day 1, t(11) = 3.16, p = 0.009 and post-TTE on Day 21 t(11) = 4.28, p = 0.001. The Caffeine group also showed a significant increase in sAA on Day 21 post-TTE relative to Day 21 baseline (Day 21 Test 1) t(11) = 4.85, p = 0.001. Similarly, the Placebo group showed a significant increase in sAA following the TTE on Day 1 t(11) = 4.03, p = 0.002 and Day 21 t(11) = 3.29, p = 0.007. There were no significant within-group differences comparing Day 1 Time 2 with Day 21 Time 2. Independent-sample t-tests showed that the only significant group difference was between the Botanical blend group (mean = 115.55, SD = 46.64) and the Placebo group (mean = 68.53, SD = 41.56) at baseline (Day 1, Test 1), t(22) = 2.44, p = 0.02 (Table 12).

**Table 12:** Salivary Alpha Amylase. Asterisks (*) indicate significant difference from baseline (Day 1, Test 1), * = p <0.05, ** = p <0.01. Dagger (†) indicates significant difference from baseline Day 21 (Day 21, Test 1), p < 0.05. Note: Units are U/mL.

	Botanical blend		Caffeine		Placebo	
	Mean	SD	Mean	SD	Mean	SD
Day 1 Test 1	115.55	48.64	112.53	85.37	68.53	41.56
Day 1 Test 2	139.5	101.55	172.48	130.83	87.03	58.54
Day 1 post-TTE	264.73^*^	204.33	282.11^**^	203.47	218.49^**^	142.43
Day 7	121.2	84.43	141.43	106.07	98.13	69.34
Day 14	97.27	42.92	91.15	34.92	117.07	83.36
Day 21 Test 1	137.03	67.73	123.2	81.24	114.55	107.73
Day 21 Test 2	194.30^*^	129.68	166.22	171.32	110.38	71.06
Day 21 post-TTE	210.42	152.81	268.32^*^^†^	148.73	212.86^**^	162.45

## Discussion

In general, we found that the botanical blend was found to be a generally safe and a potentially effective product across mood and cognitive measures. None of the study interventions resulted in a self-reported or observed adverse event nor serious adverse events. Heart rate, blood pressure readings, and cortisol were not of concern at any testing session. The measures of anxiety were improved by some of the interventions. All NIH Toolbox measures of cognition results started and were maintained above the expected normative ratings (50 for each measure). In general, where changes within or between groups occurred, they occurred in a positive direction. TTE as the measure for physical performance did not show any performance differences across the groups. In this study, the Botanical blend, Caffeine, or Placebo was no different for impacts on TTE performance. 

Mental energy was assessed using MAPSS. The MAPSS is a self-report questionnaire that consists of three subscales for Alertness, Anxiety, and Headache. We found that relative to baseline, the Botanical blend acutely increased alertness (Day 1) and increased alertness 21 days later. This was not the case for the Caffeine or the Placebo Group. In addition, comparing the groups to each other showed that the Botanical blend group had significantly higher alertness than the Caffeine and the Placebo groups during pre-dose on treatment Day 21. It is perhaps not surprising that the Caffeine group did not show increased self-reported alertness since a previous study of 379 participants showed that caffeine did not increase alertness on the MAPSS. Results from the study suggested that the increased alertness in caffeine users is related to counteracting the overnight caffeine withdrawal in caffeine users [20]. The Anxiety subscale of the MAPSS showed that there was a self-reported decrease in anxiety in the Placebo and Caffeine groups at Day 21 while anxiety remained unchanged in the Botanical blend group. While it might seem counterintuitive that the Caffeine group would self-report a reduction in anxiety, previous work has shown that caffeine in doses similar to the current study can reduce anxiety in the long term through an increase in pre-frontal cortex dopamine release [21]. None of the product groups reported an acute or long-term change in self-reported headache. In sum, MAPSS data suggest that botanical blend increases alertness without a concomitant increase in anxiety or headache.

The PVT is a widely used neurobehavioral test and is a sensitive measure of fatigue and attention deficits in clinical and experimental contexts [22,23]. Performance on psychomotor vigilant attention tasks generally improves with increased physiological arousal [24]. Reaction time measures for the PVT in our study showed that, compared to baseline testing, the Placebo and the Caffeine groups had significantly slower reaction times after baseline testing. In addition, on Day 21 (pre-dose), the Caffeine group also had a significantly slower reaction time relative to the Placebo group. Unlike the Placebo and Caffeine groups, the Botanical blend did not show decreased reaction time performance at any session (compared to their own baseline and between groups). Measures of PVT errors showed that the Botanical blend group made fewer errors than the Caffeine group on Day 7 and Day 21 pre-dose. Combined, these findings suggest that only the Botanical blend group was able to maintain a speed-accuracy tradeoff throughout the study. The speed-accuracy tradeoff describes the well-studied phenomenon that when reactions are slower, there is high accuracy and when they are faster, there is a higher error rate [25]. These findings also indicate that the increased self-reported alertness observed in the Botanical blend group occurs without increased “jitters” (as measured via errors on the PVT).

We also tested a series of cognition domains using the NIH Toolbox Cognition testing platform [15,16]. The Flanker test measures inhibitory control and attention [26]. We found that all groups generally improved on this task after baseline. Notably, the Botanical blend group was the only group to show improvement in all three sessions after baseline. In addition, relative to baseline, the Botanical blend group had significantly greater performance relative to the Caffeine group at three testing sessions. Results for the DCCS test were less robust. The DCCS is a measure of executive function and assesses cognitive flexibility [26]. Results from this test showed a trend of the Botanical blend outperforming the Caffeine group at every session post-baseline; however, this effect was only statistically significant on Day 14. We also assessed processing speed, which is the ability to process information in a certain period of time through the Pattern Comparison Test [26]. Similar to the DCCS test, all groups generally improved their performance relative to baseline testing. Despite this general improvement in performance across groups, the Botanical blend group significantly outperformed the Placebo group on Days 14 and 21 (pre-dose). Combined, these results suggest that botanical blend generally improved cognitive functioning and that these improvements appear to be most robust after two weeks of use. The only measure to show acute improvement was the Flanker inhibition attentional control task. This result makes sense given that a previous meta-analysis showed that *Bacopa monnieri* extract robustly improves attention over other cognitive domains [10]. However, since this is the first study to measure cognitive processing in the botanical blend, it is unclear if these general improvements are due mostly to the *Bacopa monnieri* extract or the combination of the products in the botanical blend.

We did not show a change in any group on the physical performance measure (TTE test). There were no significant within- or between-group differences in running times on Day 1 or Day 21. 

Cortisol was assessed as a biological measure of stress. The Botanical blend group showed a significant increase in cortisol pre-dose on Day 21. However, these values were no longer elevated post-dose or after the TTE. The Caffeine group showed a significant decrease in cortisol post-dose on Day 1. It is important to note that the values of cortisol across sessions and groups reflect levels that are within the expected range of a no-stress/baseline testing condition [27] and the expected diurnal level [28]. sAA is a correlate of sympathetic nervous system (SNS) activation and sAA levels reflect catecholaminergic (norepinephrine) activity [29]. There is an expected increase in SNS activity in response to the stress and physical demands of the TTE test. Consistent with this, all groups had a significant increase in sAA after the first TTE test (Day 1). The Botanical blend group did not show a significant increase in sAA after the TTE on Day 21, but the Caffeine and Placebo groups did. Notably, the sAA levels of the Caffeine group increased by 61% and the Placebo group increased by 92% after the TTE, while the sAA levels of the Botanical blend only increased by 8%. Despite the lower sAA levels, there was no change in TTE performance in the Botanical blend group and there was no change in max heart rate. Since this is a preliminary study, future work will need to confirm if long-term use of botanical blend decreases the adrenal medullary stress responsivity to SNS activation during physical exercise.

One potential limitation in this study was the small sample size within each group. This is of concern because low sample sizes are thought to reduce the likelihood that the results represent a true effect [30]. Nevertheless, we are confident in the current findings. Not only were our major findings sufficiently robust as to yield statistical significance at the conventional levels, but the observed effect of the botanical blend on cognition was in general agreement with each other and with previous work [10]. Follow-up studies should consider testing a single group of the botanical blend in a longitudinal study on cognitive outcome measures. An additional limitation is that due to the requirement to perform a TTE test, our sample was limited to young, healthy adults. It is unclear what the effects of the botanical blend would be in an older or cognitively vulnerable population. However, given the proposed use of *Bocopa monnieri* extract as adjuvant treatment for Alzheimer’s disease [11,12], it is reasonable to expect that the cognitive effects would be more robust in older adults.

## Conclusions

In summary, we find that the non-caffeinated energy dietary supplement, the botanical blend, we tested increases alertness and multiple measures of cognitive performance. The effects were more robust with longer use and for attention measures. These improvements in alertness and cognitive performance were not accompanied by an increase in “jitters,” as measured by errors on the PVT. The botanical blend did not improve physical performance on a TTE test either acutely or after 21 days of supplementation. Finally, there was not the expected increase in catecholamine response after the TTE on Day 21, suggesting that long-term botanical blend use decreases the catecholamine stress response of a physical endurance task. Future work should aim to carry out a longitudinal study of the cognitive effects of the botanical blend and should also consider testing the botanical blend supplementation in an older or cognitively vulnerable population.
